# Research on Disease Prediction Method Based on R-Lookahead-LSTM

**DOI:** 10.1155/2022/8431912

**Published:** 2022-04-13

**Authors:** Hailong Chen, Mei Du, Yingyu Zhang, Chang Yang

**Affiliations:** Department of Computer Science and Technology, Harbin University of Science and Technology, Harbin, Heilongjiang 150000, China

## Abstract

Cardiovascular disease is one of the most serious diseases that threaten human health in the world today. Therefore, establishing a high-quality disease prediction model is of great significance for the prevention and treatment of cardiovascular disease. In the feature selection stage, three new strong feature vectors are constructed based on the background of disease prediction and added to the original data set, and the relationship between the feature vectors is analyzed by using the correlation coefficient map. At the same time, a random forest algorithm is introduced for feature selection, and the importance ranking of features is obtained. In order to further improve the prediction effect of the model, a cardiovascular disease prediction model based on R-Lookahead-LSTM is proposed. The model based on the stochastic gradient descent algorithm of the fast weight part of the Lookahead algorithm is optimized and improved to the Rectified Adam algorithm; the Tanh activation function is further improved to the Softsign activation function to promote model convergence; and the R-Lookahead algorithm is used to further optimize the long-term memory network model. Therefore, the long- and short-term memory network model can be better improved so that the model tends to be stable as soon as possible, and it is applied to the cardiovascular disease prediction model.

## 1. Introduction

In recent years, the global information technology revolution has ushered in a new round of development climax with the vigorous development of emerging technologies such as cloud computing, big data, artificial intelligence, and the internet of things. At the same time, it has also injected new vitality into the development and transformation of traditional industries. At present, it is gradually being used in various industries such as transportation, logistics, education, and so on. In the field of medical and health care, the continuous accumulation of medical data makes the traditional medical and health care industry gradually develop to the stage of smart medical care. At this stage, the ability of computers to process data has grown rapidly by orders of magnitude, making the application scenarios of artificial intelligence in the medical and health industry more diverse, including medical image analysis, medical record and literature analysis, auxiliary diagnosis and treatment, drug development, and disease prediction. The medical internet of things [1, 2] and many other aspects have promoted the formation of a patient-centered medical data network. With the recent explosion of artificial intelligence, machine learning as the main research method of artificial intelligence has also made great progress, with outstanding achievements in speech recognition, risk research, disease diagnosis, and so on. It has high accuracy in prediction, which also provides a new research direction for disease prediction.

The prediction model has gradually developed from the original prediction model based on expert rules to the prediction model based on statistical analysis and then to the prediction model based on machine learning, with increasing efficiency and accuracy. With the development of science and technology, prediction models for ischemic heart disease [3], liver disease prediction models [4], Bayesian-based disease prediction models [5], and acute coronary syndrome diagnosis and prediction models [6] continue to emerge., which is an early disease prediction model, mainly through the establishment of prediction models through machine learning algorithms. At this stage, researchers mainly build models to predict diseases through a machine learning algorithm, and the accuracy and prediction effect need to be improved. Reference [7] proposed a hybrid method based on random forest and multivariate adaptive regression splines to establish a disease prediction model. Reference [8] established an MNN heart disease prediction model. Reference [9] proposed a new method based on heart rate signals. A new method for CAD diagnosis is based on feature extraction. Literature [10] proposed a disease prediction model using support vector machine classification algorithm and web service framework for medical data analysis. Literature [11] proposed a disease prediction based on dynamic sampling and transfer learning model. References [12, 13] proposed a multivariate time series prediction and time series knowledge graph link prediction model based on LSTM, respectively, which achieved great advantages in terms of computational cost and prediction effect. Reference [14] proposed an online medical decision support system for predicting chronic kidney disease. Reference [15] proposed an enhanced feature-level deep convolutional neural network model. The researchers went from initially using a machine algorithm to integrating statistical models and mathematical models into machine learning models, which further improved the prediction effect of the model. Reference [16] established two models to predict a blood disease. Reference [17] constructed a deep learning-based disease prediction model based on five different types of medical data. Reference [18] combined feature selection techniques with five classifications. Algorithms are used in combination. Literature [19] proposed an adaptive signal processing method based on the Levenberg–Marquardt filter of the function link artificial neural network. Literature [20] proposed an integrated deep learning method using deep learning and feature fusion model. More and more researchers are joining the study of prediction models. In order to improve the prediction effect of the models, many people use different models to predict the same disease or use different medical data sets to build a model. This to a certain extent improves the accuracy of the model. The neural network model has gradually emerged in the field of forecasting, but the parameters of the neural network are difficult to adjust, and the selection of optimization algorithms will also cause differences in forecasting results. Many prediction models only improve the structure of the neural network and do not analyze the difference in results caused by different optimization algorithms. Even when the input data set and model structure are exactly the same, the structure obtained by choosing different optimization algorithms is not the same. In view of the problem of low model accuracy caused by different optimization algorithms, this paper integrates the gradient descent optimization algorithm and the long-short-term memory network model to establish a combined disease prediction model, helping people become more aware of their physical condition, while also providing support for doctors to intervene in patients.

The second section of this paper expounds on the theoretical basis related to the model proposed in this paper; the third section introduces the basic process and main steps of the model establishment; and the fourth section carries out the experimental analysis, mainly including the comparative experiments of optimization algorithms and the lengths of different optimization algorithms. This paper compares and analyzes the algorithms proposed in this paper from different aspects through comparison experiments of time memory network models; the fifth section summarizes the full text.

## 2. Theoretical Basis

### 2.1. Random Forest Feature Selection Algorithm

The random forest algorithm [21] uses the Gini index method to evaluate the feature importance. First, the contribution of each feature vector in the data set to each tree in the random forest is judged, and then the average value is taken, and finally, the feature vectors are compared. The size of the contribution is arranged in descending order.

The calculation formula of the Gini index is shown in the following formula *P*_*k*_:

(1) $$\begin{array}{c} \text{Gini}\left(p\right)=\sum_{k=1}^{k}p_{k}\left(1-p_{k}\right). \end{array}$$

Here, *k* represents the category and *P*_*k*_ represents the sample weight of the sample *k*.

The importance score of feature *x*_*j*_ on node *m* is shown in the following formula:

(2) $$\begin{array}{c} VIM_{jm}^{\left(\text{Gini}\right)}=GI_{m}-GI_{l}-GI_{r}. \end{array}$$

Here, *GI*_*l*_ and *GI*_*r*_ represent the Gini indices of the two new nodes before and after the split, respectively.

If the node where feature *x*_*j*_ appears in tree *i* is in set *M*, then the importance score of feature *x*_*j*_ on the *i*-th tree is shown in the following formula:

(3) $$\begin{array}{c} VIM_{ij}^{\left(\text{Gini}\right)}=\sum_{m\in M}VIM_{jm}^{\left(\text{Gini}\right)}. \end{array}$$

Assuming that there are *n* trees in the random forest, the importance score is shown in the following formula:

(4) $$\begin{array}{c} VIM_{j}^{\left(\text{Gini}\right)}=\sum_{i=1}^{n}VIM_{ij}^{\left(\text{Gini}\right)}. \end{array}$$

Finally, the obtained feature importance score is normalized, and the formula is shown in the following formula:

(5) $$\begin{array}{c} VIM_{j}=\frac{{\text{VIM}}_{j}}{\sum_{i=1}^{c}VIM_{i}}. \end{array}$$

### 2.2. LSTM Algorithm

Aiming at the long-term dependence of the convolutional neural network model, Hochreiter and Schmidhuber improved on the basis of RNN and proposed a long short-term memory networks (LSTM) model, which better solves the gradient explosion and disappearance [22].

**Figure 1:**
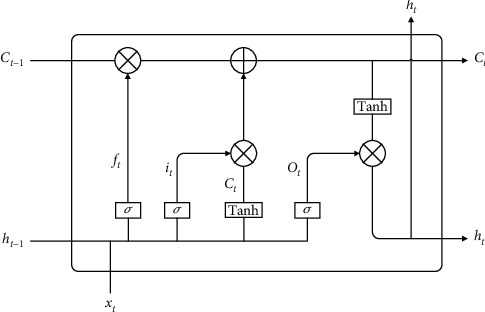
LSTM structure diagram.

Compared with the ordinary convolutional neural network model, the LSTM model increases the cell state and introduces input gates, output gates, and forget gates to save and control information. The function of the cell state is to judge whether the information is useful; the input gate determines the retention of the current information; the forget gate determines the retention of the previous state; and the output gate determines the output information according to the current state of the neural network. As shown in Figure 1, compared with the RNN model, the model has a cell state *C*_*t*_ in addition to the hidden state *h*_*t*_ at each moment. For the *t*-th time step, the forget gate, the input gate, and the output gate, respectively, represent for *f*_*t*_, *i*_*t*_, and *o*_*t*_.

The core of the LSTM model is the cell state, which consists of an activation function sigmoid and a multiplier. The sigmoid function outputs a number in the range of (0,1), indicating how much information can pass through the cell state. The processing of data by the LSTM model mainly includes the following four steps:

**Step 1 .**

Forget the door

The forget gate controls the forgetting degree of information, and the forget gate controls whether the current cell state forgets the hidden cell state at the previous moment through the output probability of the sigmoid function. The forget gate takes the state output *h*_*t-*1_ of the hidden layer at the last moment and the current input *x*_*t*_ as input and obtains the output vector *f*_*t*_ of the forget gate, which determines how much state *C*_*t-*1_ is retained by the previous neuron, so the output value *f*_*t*_ of the forget gate determines the pair of degree of retention of information. The formula expression is as follows:

(6) $$\begin{array}{c} f_{t}=\sigma \left(W_{f}\cdot \left[h_{t-1},x_{t}\right]+b_{f}\right). \end{array}$$

Here, *f*_*t*_ is the output value of the forget gate and *σ* is the sigmoid function.

**Step 2 .**

Input gate

The input gate is responsible for the input of current information, considering which new information to add to the cell state. First, the current input information is jointly determined by *h*_*t-*1_ and *x*_*t*_. Then, *h*_*t-*1_ and *x*_*t*_ get the new candidate cell state *C*_*t*_ through the Tanh network layer, and the input gate assigns weights to the components in *C*_*t*_ to control how much new information is added to the network, and its weight range is generally between (0,1). The formula expression is as follows:

(7) $$\begin{array}{c} i_{t}=\sigma \left(W_{i}\cdot \left[h_{t-1},x_{t}\right]+b_{i}\right),\\ {\tilde{C}}_{t}=\text{tach}\left(W_{c}\cdot \left[h_{t-1},x_{t}\right]+b_{c}\right). \end{array}$$

**Step 3 .**

Cell state update

Update the cell state information *C*_*t*–1_ to obtain new cell state information *C*_*t*_. The update formula is shown in the following formula:

(8) $$\begin{array}{c} C_{t}=f_{t}⊙C_{t-1}+i_{t}⊙{\tilde{C}}_{t}. \end{array}$$

Here, ⊙ is the Hadamard product.

**Step 4 .**

Output gate

Using *h*_*t*–1_ and *x*_*t*_ to calculate the current state *o*_*t*_, the new cell state *C*_*t*_ is adjusted by the Tanh activation function and multiplied by this vector, and finally, the cell output *h*_*t*_ of the current model is obtained. The formula expression is as follows:

(9) $$\begin{array}{c} o_{t}=\sigma \left(W_{o}\cdot \left[h_{t-1},x_{t}\right]+b_{o}\right),\\ h_{t}=o_{t}⊙\text{tach}\left(C_{t}\right). \end{array}$$

When training the LSTM model, there are eight sets of parameters that need to be learned by the model, namely, four sets of weights *w*_*f*_, *w*_*i*_, *w*_*o*_, and *w*_*c*_ and four sets of bias items *b*_*f*_, *b*_*i*_, *b*_*o*_, and *b*_*c*_ corresponding to *f*_*t*_, *i*_*t*_, *o*_*t*_, and *C*_*t*_. For the RNN algorithm and the LSTM algorithm, the most commonly used BPTT (back-propagation through time) algorithm [23] is used for training. There are five main steps:

  Step 1: initialize the weight parameters
  Step 2: use the above formula to forward calculate the output value of each neuron
  Step 3: calculate the error value of each neuron in reverse
  Step 4: calculate the gradient of each weight parameter according to the corresponding error value
  Step 5: use the optimization algorithm to update the weights and iterate continuously until the error converges to the specified threshold and stop training

### 2.3. Optimization

#### 2.3.1. Lookahead Algorithm

Most of the gradient descent algorithms are improved by the SGD algorithm, including the Adagrad algorithm, the Adadelta algorithm, the RMSProp algorithm, the Adam algorithm, the Nadam algorithm, and the AdaBound and AMSBound algorithms [24]. These optimization algorithms improve the exploratory training process by incorporating momentum methods or by optimizing the learning rate and finally allow the model to converge. The Lookahead algorithm [25] differs from the above algorithms in that it adopts a completely new design: two sets of weights are maintained and interleaved between them. Simply put, it allows the faster set of weights to keep exploring forward while leaving the slower set of weights behind, resulting in better long-term stability. The Lookahead algorithm flow is as follows:

  Step 1: backup the existing parameter *θ*_*t*_ of the model
  Step 2: starting from parameter *θ*_*t*_, update *k* steps with the SGD algorithm to obtain a new weight ${\tilde{\theta }}_{t}$
  Step 3: update model weights $\theta_{t}\leftarrow \theta_{t}+\alpha \left(\tilde{\theta_{t}}-\theta_{t}\right)$

The Lookahead algorithm maintains two sets of weights: fast weights and slow weights. First, an internal optimizer (generally a stochastic gradient descent optimization algorithm) is used to iteratively update the fast weights part *k* times, and then the slow weights part is updated in the direction of the last fast weights part. The fast weights part can move forward faster to better explore, and the slow weights part can play a role in maintaining stability. The two cooperate with each other to explore the entire space in a more detailed manner, enabling the Lookahead algorithm to explore faster and more stably and achieve convergence. When the fast weights part is slowly explored near the minimum value, the update of the slow weight part prompts Lookahead to explore better new regions, thereby improving the accuracy. The formulas of the fast weight part and slow weight part are as follows:

(10) $$\begin{array}{c} \text{fast weight}:\phi_{t,i+1}=\phi_{t,i}+A\left(L,\phi_{t,i+1},d\right), \end{array}$$

(11) $$\begin{array}{c} \text{slow weight: }\phi_{t+1}=\phi_{t}+\alpha \left(\phi_{t,k}-\phi_{k}\right). \end{array}$$

#### 2.3.2. RAdam Algorithm

The modified adaptive moment estimation algorithm, RAdam algorithm [26], is one of the latest optimization algorithms, which has the advantages of fast training speed and good convergence effect. Adam algorithm [27] cannot make accurate adaptive momentum selection due to lack of data in the early stage of training, the convergence speed is very fast when using the Adam algorithm to optimize, but it is easy to fall into the local optimum. Therefore, it is necessary to add a warm-up phase when the training is just started. However, the number of preheating needs to be adjusted manually, and the loss value is different for different times, and it will also be different on different data sets. Therefore, the RAdam algorithm is improved on the basis of the Adam algorithm. Based on the design of the rectifier function, the algorithm can dynamically and adaptively open or close the learning rate to adjust the gradient, which makes the initial learning rate more robust and avoids early training due to lack of the drastic change problem caused by learning enough data. The RAdam algorithm flow is as follows:

  Step 1: initialize where the step size is {*α*_*t*_}_*t*=1_^*T*^, the exponential decay rate of moment estimation is {*β*_1_, *β*_2_}, the initial parameter is *θ*_0_, the random objective function is *f*_*t*_(*θ*), the initial time step is *t* = 1, the moment of the SMA is initialized to *m*_0_=0  and *v*_0_=0, and the maximum length of the SMA is calculated as *ρ*_*∞*_=2/(1 − *β*_2_) − 1.
  Step 2: calculate the gradient *g*_*t*_ at the *t*-th step, update the second-order moment estimate *v*_*t*_, update the first-order moment estimate *m*_*t*_, and calculate *ρ*_*t*_.
  Step 3: if *ρ*_*t*_ > 4, update parameter *θ*_*t*_ by calculating adaptive learning rate and variance correction; otherwise, use nonadaptive momentum to update parameter.
  Step 4: determine whether the parameters are converged. If the parameters have converged, stop training; otherwise, let *t* = *t* + 1 and repeat steps 3 and 4 until the parameters converge.

## 3. Disease Prediction Model Based on R-Lookahead-LSTM

### 3.1. Improved LSTM Algorithm

**Figure 2:**
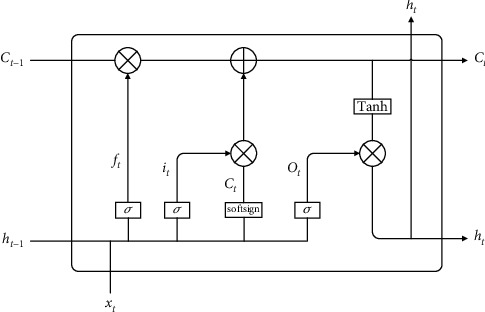
Improved LSTM model structure diagram.

In this section, the LSTM model is improved, and the Softsign activation function is used to replace the Tanh activation function in the input gate, which can make the model converge quickly. The improved LSTM model structure is shown in Figure 2.

The processing of data by the improved LSTM mainly includes the following four steps:

Step 1: control which historical information needs to be discarded by the cell state by forgetting the sigmoid activation function in the gate. The forget gate takes the state output *h*_*t*–1_ of the hidden layer at the previous moment and the current input *x*_*t*_ as input and outputs a vector *f*_*t*_, which determines how much of the state *C*_*t*–1_ of the previous neuron to retain. The formula expression is as follows:
  (12) $$\begin{array}{c} f_{t}=\sigma \left(W_{f}\cdot \left[h_{t-1},x_{t}\right]+b_{f}\right). \end{array}$$
Step 2: the input gate is responsible for the degree to which new information about the cell state is added. The information to be added first is determined by the state output *h*_*t*–1_ of the hidden layer at the previous moment and the current input *x*_*t*_. Then *h*_*t*–1_ and *x*_*t*_ get the candidate cell state *C*_*t*_ through the Softsign network layer, and the input gate assigns each component in *C*_*t*_ a weight between 0 and 1 to control how much new information the network adds. The formula expression is as follows:
  (13) $$\begin{array}{c} i_{t}=\sigma \left(W_{i}\cdot \left[h_{t-1},x_{t}\right]+b_{i}\right),\\ {\tilde{C}}_{t}=\text{Softsign}\left(W_{c}\cdot \left[h_{t-1},x_{t}\right]+b_{c}\right). \end{array}$$
Step 3: update the cell state information *C*_*t*–1_ to obtain new cell state information *C*_*t*_. The update formula is as follows:
  (14) $$\begin{array}{c} C_{t}=f_{t}*C_{t-1}+i_{t}*{\tilde{C}}_{t}. \end{array}$$
Step 4: the output gate is responsible for the determination of the output value *h*_*t*_. Use *h*_*t*–1_ and *x*_*t*_ to calculate the state *o*_*t*_ after the information passes through the output gate; *C*_*t*_ is multiplied by the vector after being adjusted by the Tanh function; and finally, the unit output of the current neural network is obtained. The formula expression is as follows:

(15) $$\begin{array}{c} o_{t}=\sigma \left(W_{o}\cdot \left[h_{t-1},x_{t}\right]+b_{o}\right),\\ h_{t}=o_{t}*\text{tach}\left(C_{t}\right). \end{array}$$

When training the LSTM model, there are eight sets of parameters that need to be learned by the model, namely, four sets of weights *w*_*f*_, *w*_*t*_, *w*_*o*_, and *w*_*c*_ and four sets of bias items *b*_*f*_, *b*_*i*_, *b*_*o*_, and *b*_*c*_ corresponding to *f*_*t*_, *i*_*t*_, *o*_*t*_, and *C*_*t*_. The training process is as follows:

  Step 1: initialize the weight parameters
  Step 2: use the above formula to forward calculate the output value of each neuron
  Step 3: calculate the error value of each neuron in reverse
  Step 4: calculate the gradient of each weight parameter according to the corresponding error value
  Step 5: use the R-Lookahead optimization algorithm to update the weights and iterate continuously until the error converges to the specified threshold and stop training

### 3.2. Improved Lookahead Algorithm

To solve LSTM, it is necessary to continuously solve and update various weights and bias terms of the model to make it approach or reach the optimal value, so as to achieve the effect of minimizing the loss function. This chapter optimizes the LSTM model using the improved Lookahead optimization algorithm (R-Lookahead algorithm). The Lookahead algorithm maintains two sets of weights, including the fast weights part and the slow weights part. This section makes improvements to the fast weights section, using a modified adaptive moment estimation algorithm to adjust the gradient. The RAdam algorithm can dynamically switch on and off the adaptive momentum so that the entire training process can be quickly stabilized, avoiding the phenomenon of violent oscillation caused by the limited number of samples in the early training period. The R-Lookahead algorithm is shown in Algorithm 1.

### 3.3. R-Lookahead-LSTM Disease Prediction Model

**Figure 3:**
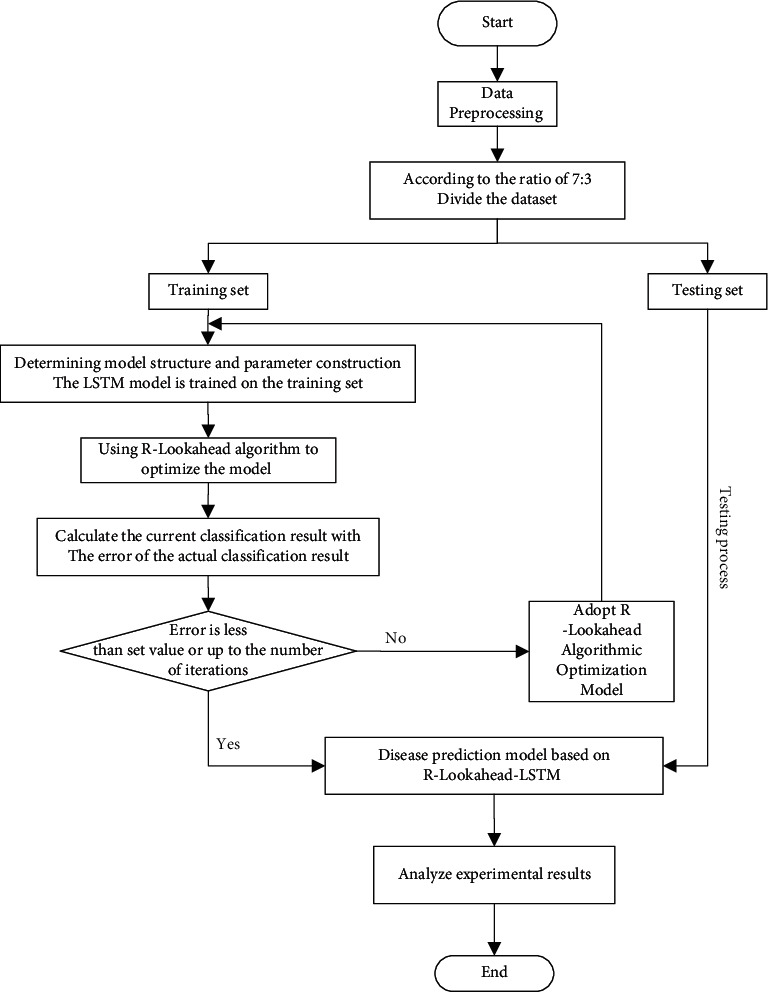
Flow chart of a disease prediction model based on R-Lookahead-LSTM.

As the main algorithm of this section, the LSTM algorithm can solve the problem of gradient explosion and disappearance in the RNN model training process to a certain extent. The model structure of the LSTM algorithm is optimized and improved on the basis of the model structure of the RNN algorithm. This algorithm sets an output gate, an input gate, and a forget gate in each neuron of the RNN algorithm. For data samples, on the one hand, the LSTM algorithm improves the long-term dependence of the RNN algorithm to a certain extent. On the other hand, the Softsign activation function in the improved LSTM algorithm can improve the convergence speed of the model and make the model stabilize as soon as possible. However, due to the overall structure of the neural network model, no matter how it is optimized and reconstructed, in some application scenarios, its training results will still appear to be more or less locally optimal. In view of the above problems, this chapter uses the R-Lookahead algorithm to optimize and improve the LSTM model and proposes the R-Lookahead-LSTM model for predicting cardiovascular disease. The workflow of the R-Lookahead-LSTM model is shown in Figure 3, and the model building process is shown in Algorithm 2.

## 4. Empirical Analysis

### 4.1. Data Preprocessing

The cardiovascular disease data set selected in this paper includes three types of data: objective facts, examination results during physical examination, and information provided by patients, with 11 input variables and 1 target variable. This paper aims to predict whether a sample has cardiovascular disease through body-related features. If the sample has cardiovascular disease, the label is 1, and if the sample is healthy, the label is 0. This data set is a classic two-category data set, which meets the requirements for data sets in this paper.

#### 4.1.1. Construction of Strong Eigenvectors

The purpose of this paper is to build a predictive model suitable for cardiovascular disease. If the input of the model only relies on the feature vector in the data set, there is a lack of strong features in the context of the prediction of the disease, and the prediction result is not ideal. In order to generate new strong features, based on the feature-derived method, this paper analyzes the existing feature vectors in the data set, mines the underlying laws and data structures of cardiovascular disease prediction problems, and constructs a series of new features. The process of constructing new features will be described in detail below.

**Figure 4:**
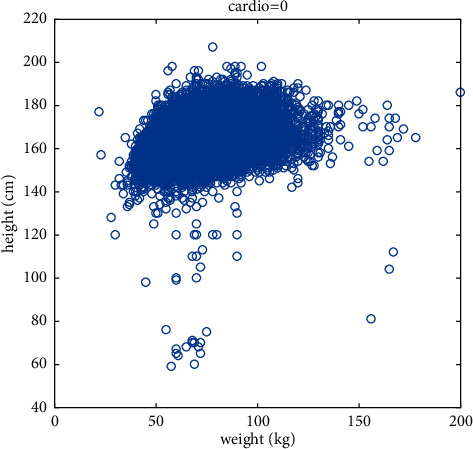
Scatter diagram of the relationship between height, weight, and healthy samples.

**Figure 5:**
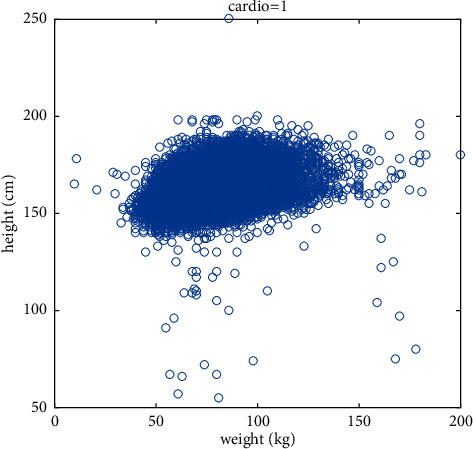
Scatter diagram of the relationship between height, weight, and sick samples.

Aiming at the prediction of cardiovascular disease, this paper firstly selects height and weight for targeted research, analyzes the relationship between height and weight and whether or not the disease is diagnosed, and constructs new features based on their relationship. Figures 4 and 5 are scatter plots of height, weight, and disease, depicting the distribution of height and weight in the cardiovascular disease data set, which can reflect the relationship between height, weight, and disease, as well as the overall law. As you can see from the graph, for the same height, people with heavier weights are more likely to have the disease. Further analysis, obesity may be an important factor leading to cardiovascular disease. In order to make the prediction model fit this point more accurately, a new eigenvector body health index (BMI) is added to comprehensively consider height and weight, and the calculation formula is shown in the following formula:

(16) $$\begin{array}{c} \text{BMI}=\frac{\text{weight}}{{\text{height}}^{2}}\times 100\%. \end{array}$$

Mean arterial pressure (MAP) is the mean arterial blood pressure level throughout a cardiac cycle. Pulse pressure (PP) is the pressure difference between ap_hi and ap_lo. Studies have shown that MAP and PP may be risk factors for cardiovascular disease. In order to make the predictive ability of the model more accurate, new eigenvectors mean arterial pressure (MAP) and pulse pressure (PP) are added, and the calculation formulas are shown in the following formula:

(17) $$\begin{array}{c} \text{MAP}=\frac{ap\_hi+2ap\_lo}{3}, \end{array}$$

(18) $$\begin{array}{c} PP=ap\_hi-ap\_lo. \end{array}$$

#### 4.1.2. Data Processing

Due to the huge amount of data in the cardiovascular disease data set and the nonuniform type of features, which makes the research work difficult, the data needs to be processed first. This paper checks the duplicate values. After checking, it is found that there are 24 duplicate samples in the data set. The duplicate items have no effect on the training of the model, so the duplicate samples are deleted. A box plot was used to detect outliers. Looking closely at the data, we noticed that the youngest was about 29 years old, the youngest was 55 cm tall, the youngest weighed 10 kg, the tallest was 250 cm tall, and the heaviest weighed 200 kg, the smallest ap_hi is –150, and the smallest ap_lo is –70. In order to deal with such outliers, we delete samples with height and weight below 5% or above 95% and delete samples with negative blood pressure. The normal value for diastolic blood pressure is 60–80 mmHg, and the normal value for systolic blood pressure is 90–120 mmHg, so this paper will remove ap_hi outliers over 200 and ap_lo outliers less than 50. For the cholesterol and blood sugar fields, this subsection takes the form of the data set itself because the progression of the data also represents the level of the sample in this feature. At this point, the data set has been basically processed in this section, but there are still two problems. One is that the continuous variables and the categorical variables in the features do not belong to the same dimension. If the original data values are used directly, the features will affect the prediction results. The degree of influence is different, and the features are not comparable; second, some features are in the form of categorical variables, and the numerical size will mislead the model to a certain extent, so the data needs to be normalized and one-hot coding. In addition, in order to more intuitively see the relationship between features, this paper uses the Pearson correlation coefficient to draw a heat map.

*(1) Normalizing*. In large-scale data analysis projects, data often have different sources, and their dimensions and specifications are different and cannot be directly compared, so normalization processing is required to eliminate the resulting bias. After the original data is normalized, all indicators are in the same order of magnitude, and the input features and target predicted values approximately obey the normal distribution, which helps eliminate outliers and noise in the data and is suitable for comprehensive comparative evaluation. This section normalizes the “age,” “height,” “weight,” “zap_hi,” “ap_lo,” “MAP,” and “PP” fields to reduce the feature data of different magnitudes covering other features on the target effect of the function. The normalization formula is as follows:

(19) $$\begin{array}{c} y_{i}=\frac{x_{i}-\underset{1<i<n}{\min\left(x_{i}\right)}}{\underset{1<i<n}{\max\left(x_{i}\right)}-\underset{1<i<n}{\min\left(x_{i}\right)}}. \end{array}$$

*(2) One-Hot Encoding*. In machine learning algorithms, we often encounter categorical features. These feature values are not continuous but discrete and disordered, so it is necessary to digitize such data. Plotting the height distribution by violin drawing, we found that the height of class 2 is always higher than that of class 1, so class 1 represents women and class 2 represents men, and 2 is always numerically higher than 1, which will mislead the model to some extent, so the gender field uses one-hot encoding in this paper.

*(3) Heat Map*. According to the size of the correlation coefficient corresponding to the different square colors in the heat map, the size of the correlation between the variables can be judged, and the relationship between the various features can be observed more intuitively. The calculation formula is as follows:

(20) $$\begin{array}{c} \rho_{X_{1}X_{2}}=\frac{\text{Cov}\left(X_{1},X_{2}\right)}{\sqrt{DX_{1},DX_{2}}}=\frac{EX_{1}X_{2}-EX_{1}*EX_{2}}{\sqrt{DX_{1}*DX_{2}}}, \end{array}$$

where *ρ* 1 is the correlation coefficient, Cov is the covariance, and *E* is the mathematical expectation.

**Figure 6:**
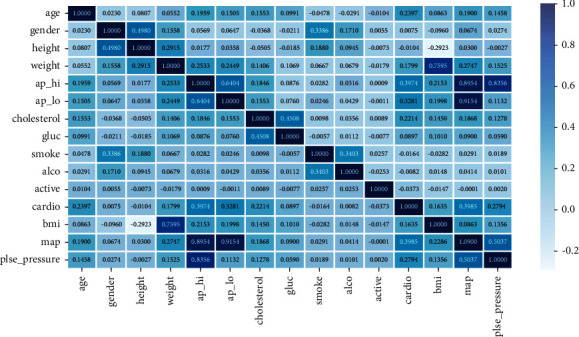
Correlation diagram of characteristic variables.

From Figure 6, we can easily see the relationship between data and data, which lays the foundation for subsequent feature selection.

### 4.2. Feature Selection

After feature construction and data processing, this paper uses a random forest algorithm to model all samples for feature selection. This article uses the Scikit-learn library in Python. First, load the RandomForestClassifier module in the Scikit-learn library to model all the data. When the model is built, we use the feature_importances_ function in the module to output the importance of all features. This function will calculate and record the Gini coefficient change value of each node after feature splitting during the tree construction process. Finally, a unified normalization can be done to obtain the importance of each feature. The feature importance rankings are shown in Table 1.

Through the feature importance ranking made by the random forest feature selection algorithm, several important information can be found. First, the three new feature vectors MAP, PP, and BMI constructed in this paper are in the relatively high position, which shows that the constructed new feature vector plays a very important role in the prediction of cardiovascular disease; second, the feature importance ranking made by the random forest feature selection algorithm is basically the same as the result of the heat map, which shows that the important features selected by the random forest algorithm are very important. It is very convincing. In this paper, the top 12 features are selected as the input vector of the cardiovascular disease prediction model constructed in this paper. Removing the features with lower rankings through feature selection can speed up the running speed of the next prediction algorithm and at the same time improve the prediction accuracy of the obtained model and improve the model's overall performance.

### 4.3. Metric Indicator

#### 4.3.1. Confusion Matrix

The confusion matrix is a metric for judging the results of a classification model and is part of model evaluation. Confusion matrices are mostly used to judge the pros and cons of classifiers and are suitable for classified data models. The confusion matrix is shown in Table 2.

True positive (TP) and true negative (TN) measure the ability of a classification model to predict whether a patient has a disease or not, and false positive (FP) and false negative (FN) identify the number of false predictions produced by the predictive model. The accuracy rate represents the overall predictive ability of the machine learning model and is used to measure the success of the predictions of the disease prediction model. The recall is used to measure the sensitivity of a disease prediction model with the aim of recalling potential cases. F-score is the weighted harmonic average of precision and recall, which is often used to evaluate the quality of classification models. F1 combines the results of precision and recall, and when it is higher, it can indicate that the experimental method is more effective. This paper uses different performance indicators to evaluate the model proposed in this paper, and the formula is shown in Table 3.

#### 4.3.2. MCC Value

The Matthews correlation coefficient (MCC value) [28] is a contingency matrix method for calculating the Pearson product-moment correlation coefficient between actual and predicted values. The key advantage of the Matthews correlation coefficient is that the classifier must make correct predictions for most negative cases and most positive cases to get a high-quality score, independent of their proportion in the entire data set. The formula for calculating the MCC value is as follows:

(21) $$\begin{array}{c} \text{MCC}=\frac{\text{TP}*\text{TN}-\text{FP}*\text{FN}}{\sqrt{\left(\left(\text{TP}+\text{FP}\right)*\left(\text{TP}+\text{FN}\right)*\left(\text{TN}+\text{FP}\right)*\left(\text{TN}+\text{FN}\right)\right)}}. \end{array}$$

### 4.4. Analysis of Results

#### 4.4.1. Optimization Algorithm Experiment Comparison

In order to show the advantages of the improved Lookahead algorithm proposed in this paper, RMSprop algorithm [29], Adam algorithm, RAdam algorithm, and Lookahead algorithm are used as comparison algorithms, and the parameters are adjusted for comparison experiments. The parameters of each optimization algorithm are set as shown in Table 4.

**Figure 7:**
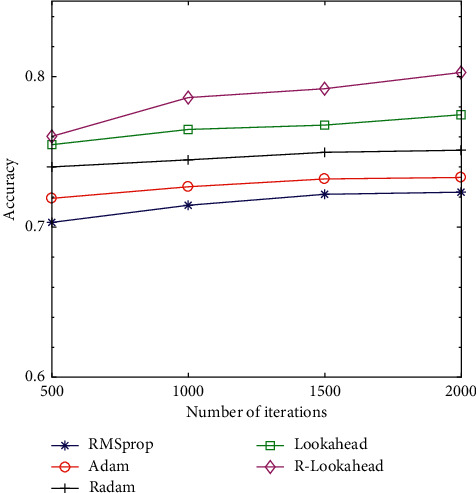
Accuracy graph of optimization algorithm.

**Figure 8:**
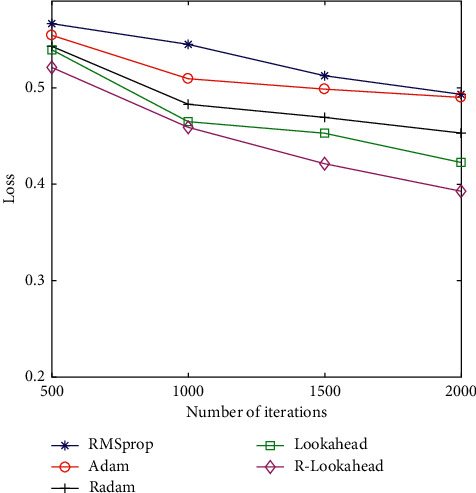
The loss graph of the optimized algorithm.

In order to compare the performance of the above optimization algorithms, this section tests the RMSprop, Adam, RAdam, Lookahead, and R-Lookahead algorithms and uses accuracy and loss as evaluation indicators. The accuracy graph and loss graph of the optimization algorithm are shown in Figures 7 and 8. The results show that with the increase of the number of iterations, the correct rate of each optimization algorithm keeps increasing, and the loss value keeps decreasing. Among them, the RAdam algorithm has been improved on the basis of Adam. It can dynamically open or close the adaptive learning rate to adjust the gradient according to the variance of the adaptive rate. Therefore, when the number of iterations is 500, the accuracy and loss are better than Adam's algorithm. The Lookahead algorithm maintains two sets of weights to achieve faster convergence. At this time, the traditional Lookahead algorithm slow weight uses the SGD algorithm, which also achieves good results. The accuracy is 0.7747, and the loss is 0.4226. When the SGD algorithm in the slow weight part of the Lookahead algorithm is improved to RAdam, this improves the speed to a certain extent and is more stable, and the loss value is also smaller to 0.3928. The specific results of the experiment are shown in Tables 5 and 6.

#### 4.4.2. Comparison of LSTM Models of Different Optimization Algorithms

**Figure 9:**
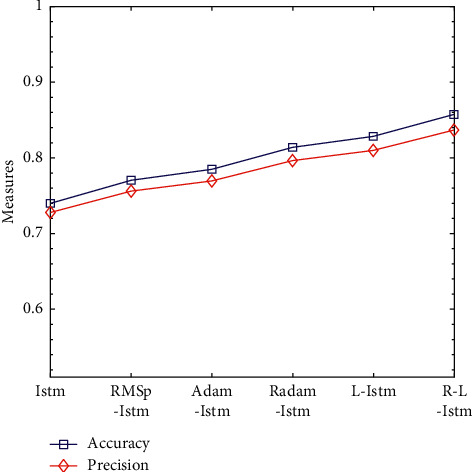
Accuracy and precision line graphs of LSTM models under different optimization algorithms.

**Figure 10:**
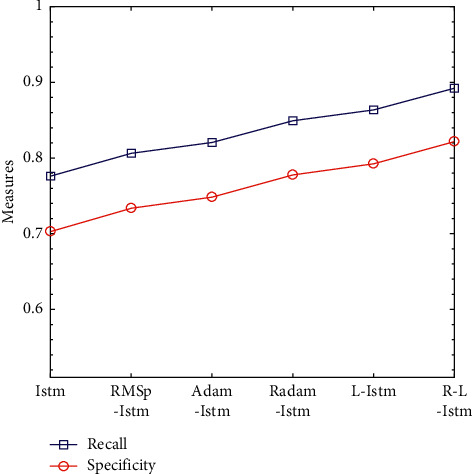
Recall rate and specificity line graphs of LSTM models under different optimization algorithms.

**Figure 11:**
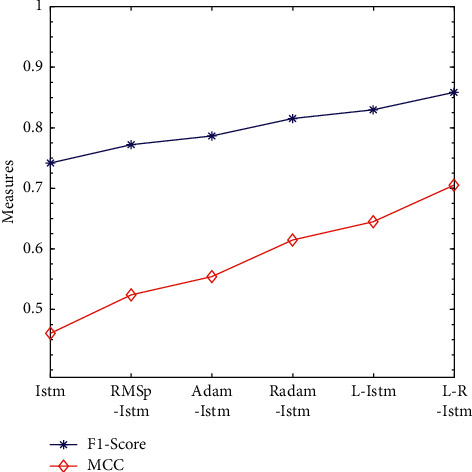
Line graph of *F*1-Score and MCC values of LSTM models under different optimization algorithms.

The optimization problem is one of the most important research contents in the field of machine learning. Even when the model structure and model input data are exactly the same, the results obtained by selecting different deep learning optimization algorithms are different. In order to better compare the effects of RMSprop, Adam, Radam, Lookahead, and R-Lookahead algorithms on the LSTM model, this section integrates the above optimization algorithms with the models established in this section and analyzes LSTM models using different algorithms in detail. Experiments show that the Lookahead algorithm and the Lookahead algorithm using RAdam have achieved good results, with the correct rate being 0.828296 and 0.857287, respectively; the traditional LSTM model has a correct rate of 0.739870 and a recall rate of 0.776074; the R-Lookahead-LSTM model proposed in this chapter has a recall rate of 0.892120 and an MCC value of 0.715926, which indicates that the cardiovascular disease prediction model established in this chapter has good generalization ability and can make correct predictions for most positive and negative situations. The experimental results are shown in Table 7 and Figures 9–11.

## 5. Summary

The disease prediction model is proposed to help doctors make decisions more informatively and accurately. In order to scientifically and effectively assist doctors in medical decision-making, this paper proposes the R-Lookahead-LSTM model for disease prediction. In order to better highlight the prediction effect of the model proposed in this paper, this study compares the proposed model with LSTM, RMSprop-LSTM, Adam-LSTM, RAdam-LSTM, and Lookahead-LSTM in detail. Accuracy, recall, *F*1-score, MCC value, and other aspects of the prediction model were compared and analyzed. Compared with the traditional LSTM model, the accuracy rate of the proposed model increased by 0.117417; the precision rate increased by 0.108828; the recall rate increased by 0.116046; the *F*1-score increased by 0.112320; and the MCC value increased by 0.235585. And MCC values increased significantly. For a disease prediction model, obtaining a good accuracy rate indicates that the model has a good classification effect; obtaining a relatively high recall rate, that is, recalling possible case samples, has very important practical significance. The MCC value shows that the model proposed in this section can make correct predictions for most negative cases and most positive cases and achieves the desired effect. The whole experimental process clearly shows that the model proposed in this chapter has good classification performance and achieves the desired effect, providing a new method for the field of disease prediction.

## Figures and Tables

**Algorithm 1:**
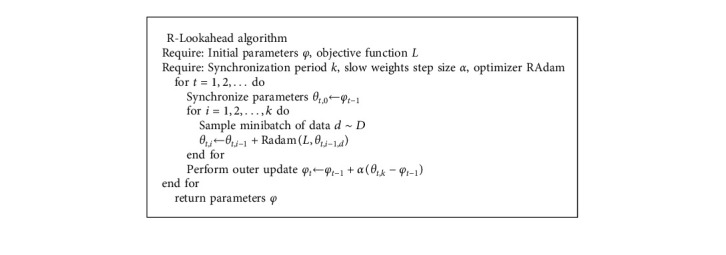
R-Lookahead algorithm.

**Algorithm 2:**
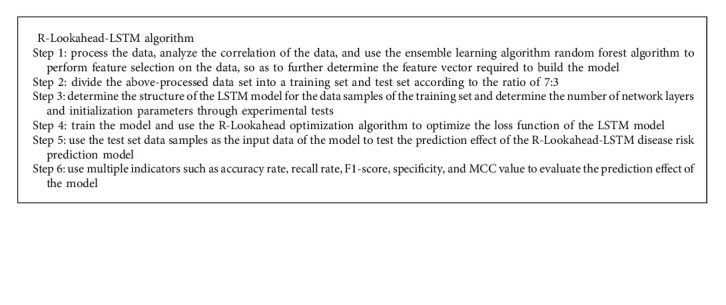
R-Lookahead-LSTM algorithm.

**Table 1:** Ranking of feature importance.

Ranking	Feature	Feature importance coefficient
1	MAP	0.260602
2	ap_hi	0.155965
3	ap_lo	0.116014
4	PP	0.110476
5	age	0.094877
6	cholesterol	0.077568
7	weight	0.048473
8	BMI	0.038585
9	gluc	0.036257
10	smoke	0.016611
11	active	0.015657
12	alco	0.013322
13	gender	0.008640
14	height	0.006953

**Table 2:** Confusion matrix.

Confusion matrix	Predict positive class	Predict negative class
Actual positive class	TP	FN
Actual negative class	FP	TN

**Table 3:** Performance indicators.

Name	Formula
Accuracy	Accuracy=TP+TN/TP+TN+FP+FN
Precision	Precision=TP/TP+FP
Recall	Recall=TP/TP+FN
Specificity	Specificity=TN/TN+FP
*F*1-score	*F*1 − score=2precision*∗*recall/precision+recall

**Table 4:** Optimization algorithm parameter settings.

Optimizers	learning_rate	beta_1	beta_2	Epsilon	*γ*	*k*	*α*
RMSprop	0.001				0.9		
Adam	0.001	0.9	0.999	1*e* − 08			
RAdam	0.0001						
Lookahead						5	0.5
R-Lookahead						5	0.5

**Table 5:** Accuracy comparison of the optimization algorithms.

Optimizers	Accuracy			
	500	1,000	1,500	2,000
RMSprop	0.7031	0.7145	0.7218	0.7232
Adam	0.7191	0.7268	0.7320	0.7330
RAdam	0.7400	0.7447	0.7497	0.7511
Lookahead	0.7548	0.7649	0.7678	0.7747
R-Lookahead	0.7603	0.7861	0.7920	0.8028

**Table 6:** Loss comparison of optimization algorithms.

Optimizers	Loss			
	500	1,000	1,500	2,000
RMSprop	0.5655	0.5450	0.5125	0.4932
Adam	0.5544	0.5094	0.4986	0.4900
RAdam	0.5430	0.4829	0.4693	0.4531
Lookahead	0.5394	0.4639	0.4529	0.4226
R-Lookahead	0.5211	0.4590	0.4213	0.3928

**Table 7:** Comparison of LSTM models with different optimization algorithms.

Classifiers	Measures					
	Accuracy	Precision	Recall	*F*1-score	Specificity	MCC
LSTM	0.739870	0.727797	0.776074	0.751161	0.702801	0.480341
RMSp-LSTM	0.770312	0.756012	0.806160	0.780281	0.733607	0.541419
Adam-LSTM	0.784808	0.769447	0.820487	0.794148	0.748276	0.570503
RAdam-LSTM	0.813800	0.796318	0.849140	0.821881	0.777614	0.628672
Lookahead-LSTM	0.828296	0.809754	0.863467	0.835748	0.792283	0.657757
R-Lookahead-LSTM	0.857287	0.836625	0.892120	0.863481	0.821622	0.715926

## Data Availability

In this paper, we used the cardiovascular disease data set. The cardiovascular disease data set can be obtained from the following website: https://www.kaggle.com/sulianova/cardiovascular-disease-dataset#cardio_train.csv.
